# Luxation traumatique de la hanche chez l'enfant (à propos d'un cas)

**DOI:** 10.11604/pamj.2014.19.397.5713

**Published:** 2014-12-22

**Authors:** Ismail Hmouri, Mohamed Saleh Berrada

**Affiliations:** 1 Clinique de Chirurgie Traumatologique et Orthopédique, CHU Avicenne, Rabat, Maroc

**Keywords:** Luxation, hanche, enfant, dislocation, hip, child

## Image en medicine

Il s'agit d'un enfant de 5ans, sans antécédent pathologique notable, victime d'une chute de sa hauteur à la cour de l’école, occasionnant chez lui une douleur et une impotence fonctionnelles totales du membre inférieur gauche. L'examen clinique trouve un membre inférieur raccourci en adduction et rotation interne. Le bilan radiologique montre une luxation postérieure pure de la hanche sans lésion associée (A). La prise en charge en urgence a consisté en une réduction de la luxation sous anesthésie générale (B), suivie de la mise en place d'une traction non collée pendant 3 semaines.

**Figure 1:**
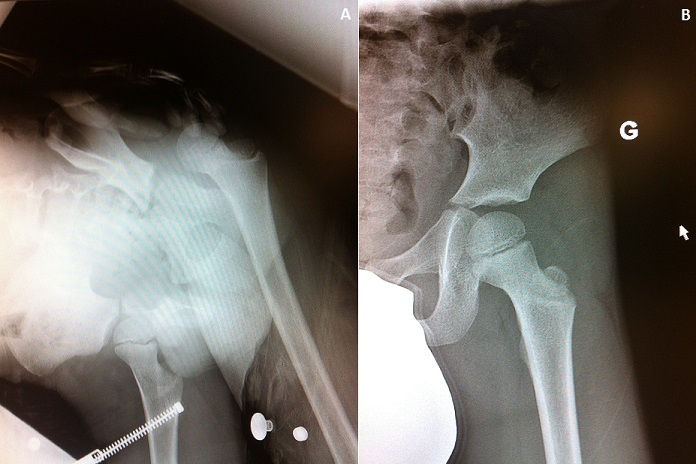
(A) radiographie du bassin montrant la luxation postérieure de la hanche; (B): radiographie du bassin de contrôle montrant la réduction de la luxation

